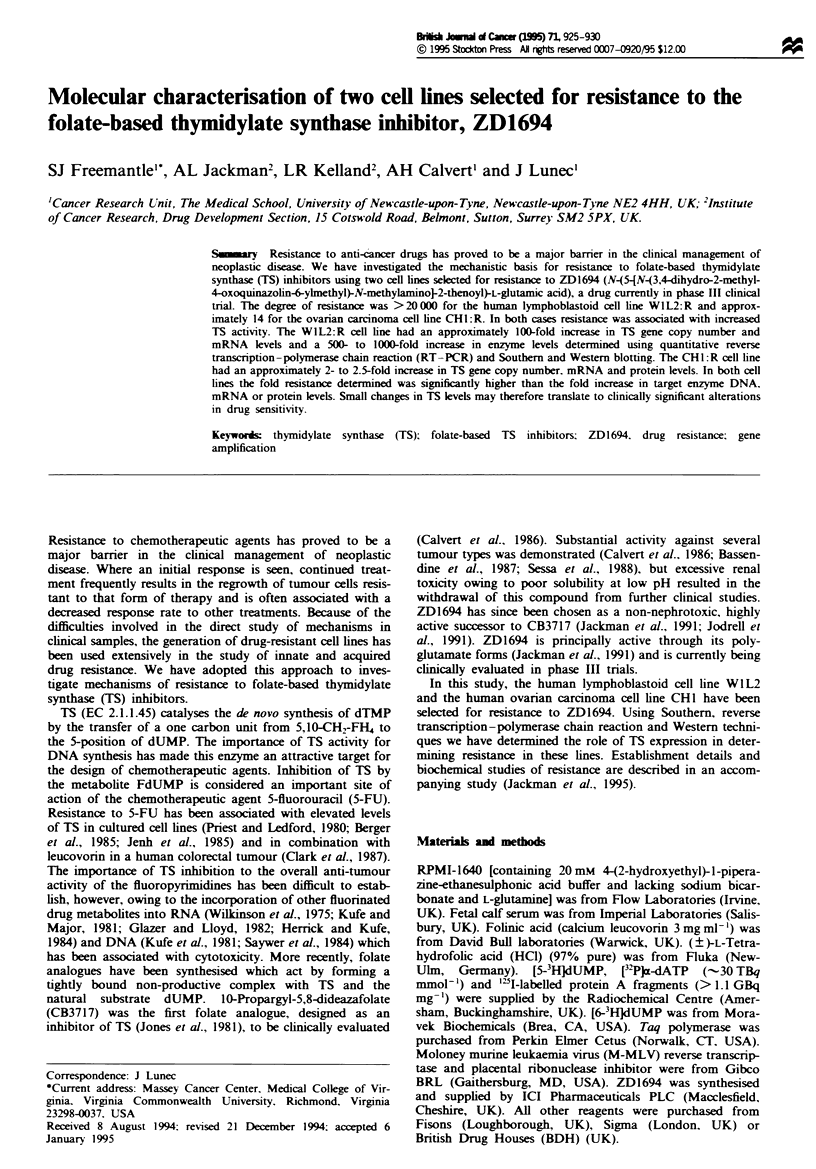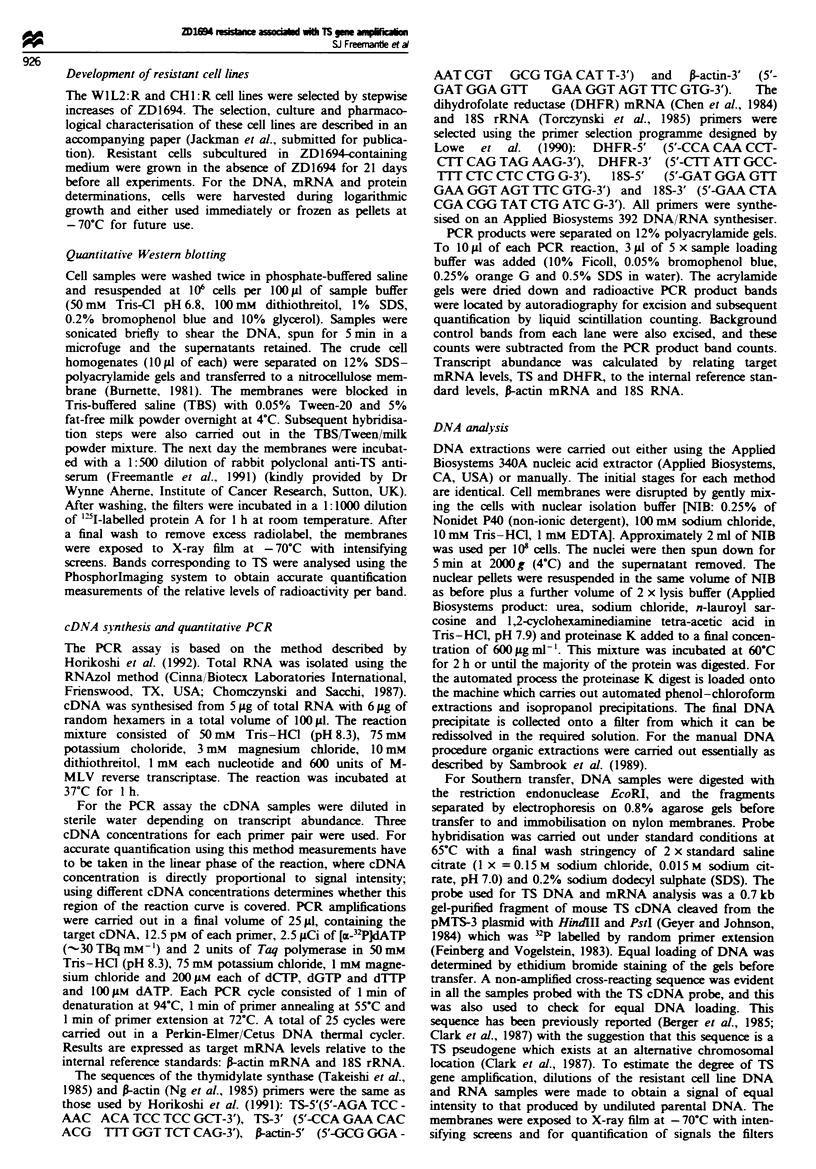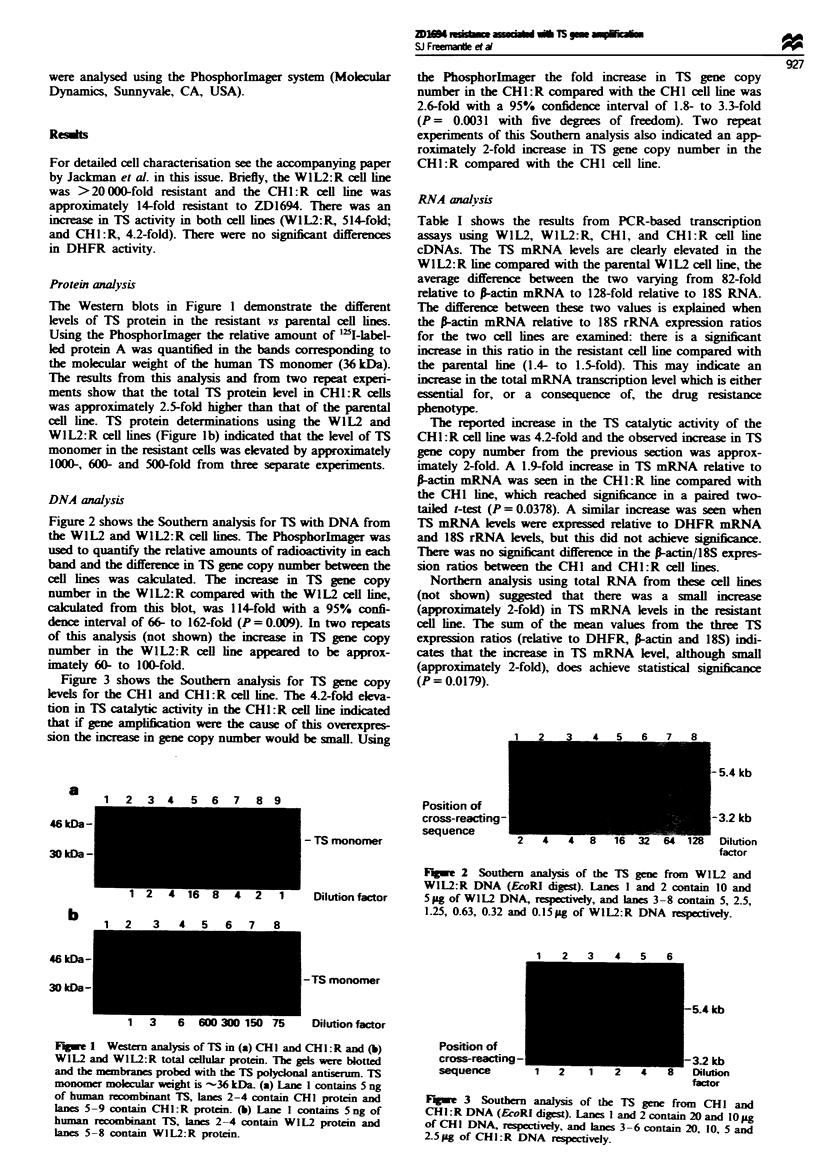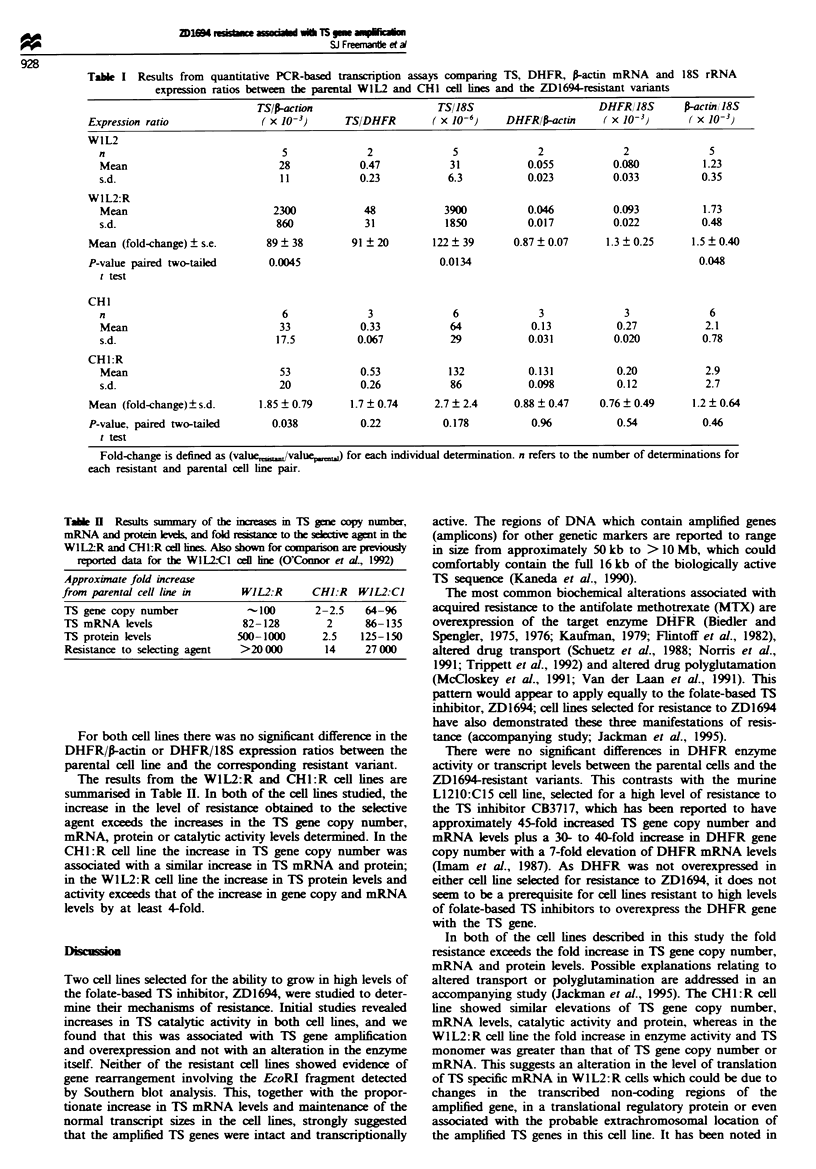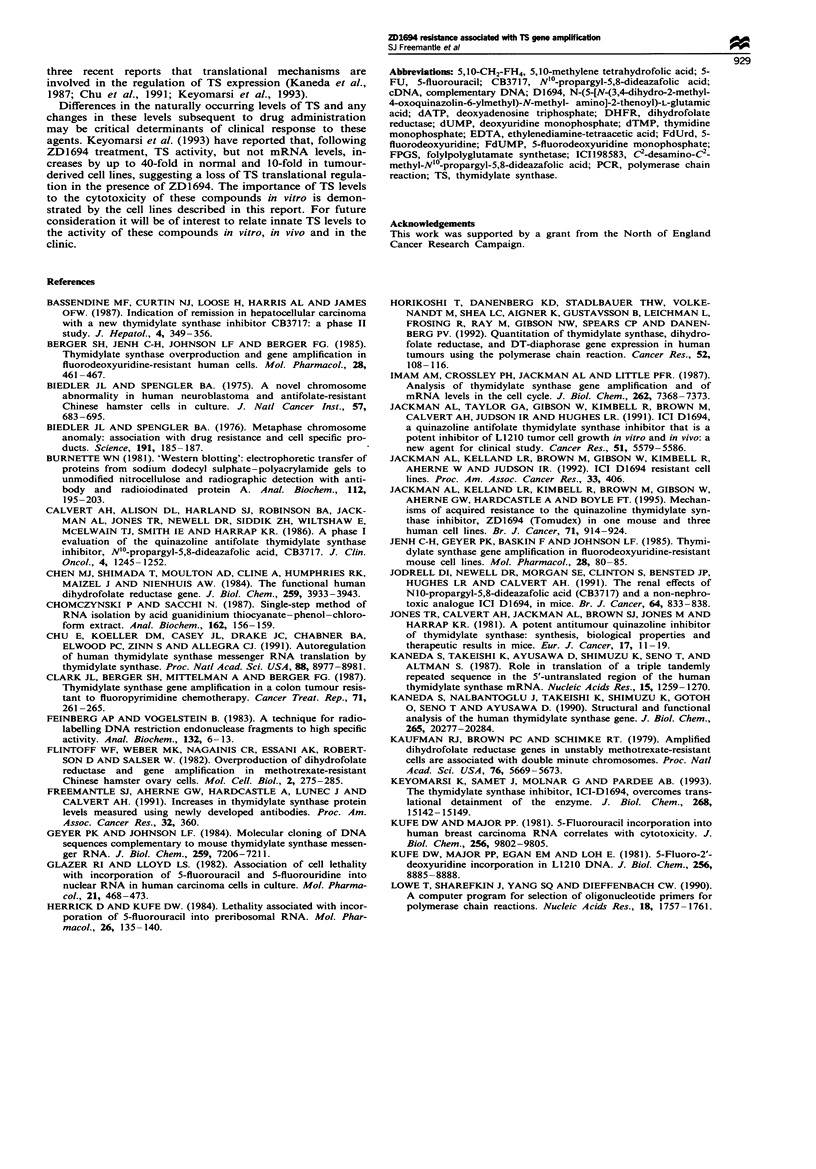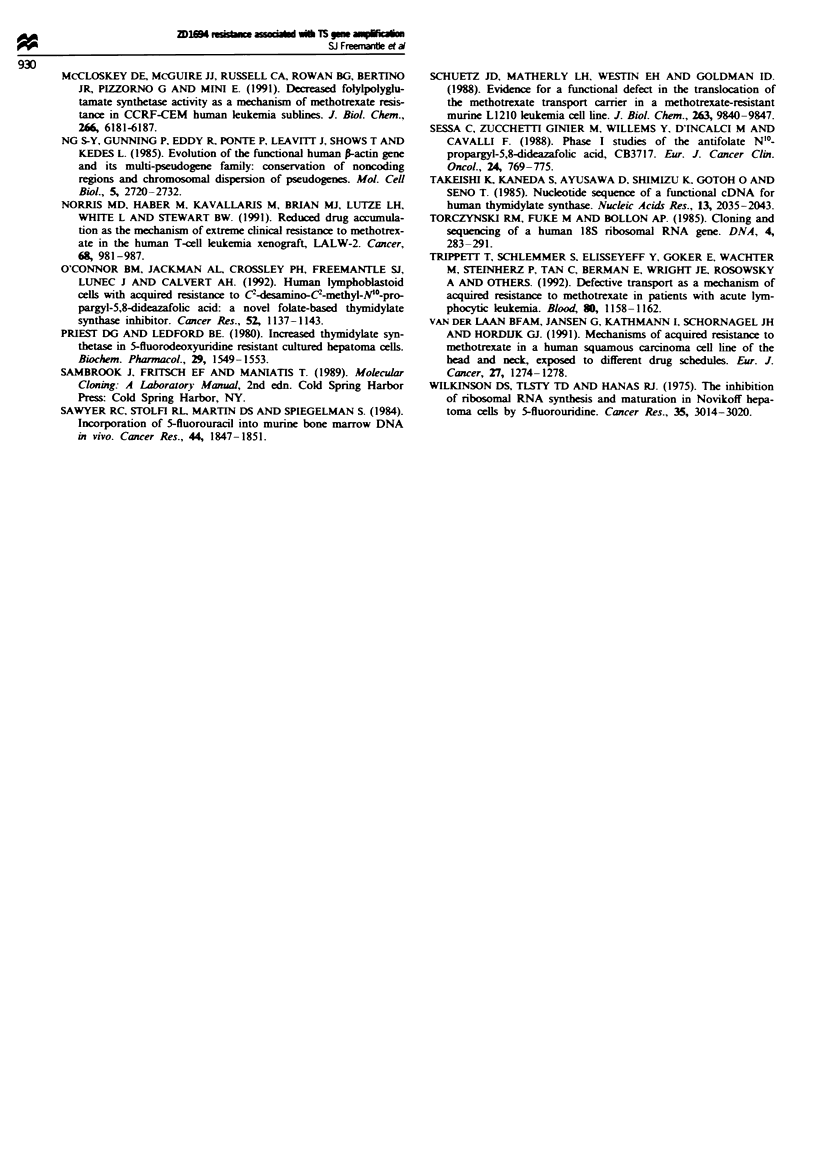# Molecular characterisation of two cell lines selected for resistance to the folate-based thymidylate synthase inhibitor, ZD1694.

**DOI:** 10.1038/bjc.1995.179

**Published:** 1995-05

**Authors:** S. J. Freemantle, A. L. Jackman, L. R. Kelland, A. H. Calvert, J. Lunec

**Affiliations:** Cancer Research Unit, Medical School, University of Newcastle-upon-Tyne, UK.

## Abstract

**Images:**


British Journal of Cancer (1995) 71, 925-930

© 1995 Stockton Press All rights reserved 0007-0920/95 $12.00

### Molecular characterisation of two cell lines selected for resistance to the folate-based thymidylate synthase inhibitor, ZD1694

SJ Freemantle[1*], AL Jackman[2], LR Kelland[2], AH Calvert[1] and J Lunec[1]

[1]*Cancer Research Unit, The Medical School, University of Newcastle-upon-Tyne, Newcastle-upon-Tyne NE2 4HH, UK;* [2]*Institute of Cancer Research, Drug Development Section, 15 Cotswold Road, Belmont, Sutton, Surrey SM2 5PX, UK.*

**Summary** Resistance to anti-cancer drugs has proved to be a major barrier in the clinical management of neoplastic disease. We have investigated the mechanistic basis for resistance to folate-based thymidylate synthase (TS) inhibitors using two cell lines selected for resistance to ZD1694 (*N*-(5-[*N*-(3,4-dihydro-2-methyl-4-oxoquinazolin-6-ylmethyl)-*N*-methylamino]-2-thenoyl)-L-glutamic acid), a drug currently in phase III clinical trial. The degree of resistance was >20 000 for the human lymphoblastoid cell line W1L2:R and approximately 14 for the ovarian carcinoma cell line CH1:R. In both cases resistance was associated with increased TS activity. The W1L2:R cell line had an approximately 100-fold increase in TS gene copy number and mRNA levels and a 500- to 1000-fold increase in enzyme levels determined using quantitative reverse transcription-polymerase chain reaction (RT-PCR) and Southern and Western blotting. The CH1:R cell line had an approximately 2- to 2.5-fold increase in TS gene copy number, mRNA and protein levels. In both cell lines the fold resistance determined was significantly higher than the fold increase in target enzyme DNA, mRNA or protein levels. Small changes in TS levels may therefore translate to clinically significant alterations in drug sensitivity.

**Keywords:** thymidylate synthase (TS); folate-based TS inhibitors; ZD1694, drug resistance; gene amplification

Resistance to chemotherapeutic agents has proved to be a major barrier in the clinical management of neoplastic disease. Where an initial response is seen, continued treatment frequently results in the regrowth of tumour cells resistant to that form of therapy and is often associated with a decreased response rate to other treatments. Because of the difficulties involved in the direct study of mechanisms in clinical samples, the generation of drug-resistant cell lines has been used extensively in the study of innate and acquired drug resistance. We have adopted this approach to investigate mechanisms of resistance to folate-based thymidylate synthase (TS) inhibitors.

TS (EC 2.1.1.45) catalyses the *de novo* synthesis of dTMP by the transfer of a one carbon unit from 5,10-$CH_2$-$FH_4$ to the 5-position of dUMP. The importance of TS activity for DNA synthesis has made this enzyme an attractive target for the design of chemotherapeutic agents. Inhibition of TS by the metabolite FdUMP is considered an important site of action of the chemotherapeutic agent 5-fluorouracil (5-FU). Resistance to 5-FU has been associated with elevated levels of TS in cultured cell lines (Priest and Ledford, 1980; Berger *et al.*, 1985; Jenh *et al.*, 1985) and in combination with leucovorin in a human colorectal tumour (Clark *et al.*, 1987). The importance of TS inhibition to the overall anti-tumour activity of the fluoropyrimidines has been difficult to establish, however, owing to the incorporation of other fluorinated drug metabolites into RNA (Wilkinson *et al.*, 1975; Kufe and Major, 1981; Glazer and Lloyd, 1982; Herrick and Kufe, 1984) and DNA (Kufe *et al.*, 1981; Saywer *et al.*, 1984) which has been associated with cytotoxicity. More recently, folate analogues have been synthesised which act by forming a tightly bound non-productive complex with TS and the natural substrate dUMP. 10-Propargyl-5,8-dideazafolate (CB3717) was the first folate analogue, designed as an inhibitor of TS (Jones *et al.*, 1981), to be clinically evaluated (Calvert *et al.*, 1986). Substantial activity against several tumour types was demonstrated (Calvert *et al.*, 1986; Bassendine *et al.*, 1987; Sessa *et al.*, 1988), but excessive renal toxicity owing to poor solubility at low pH resulted in the withdrawal of this compound from further clinical studies. ZD1694 has since been chosen as a non-nephrotoxic, highly active successor to CB3717 (Jackman *et al.*, 1991; Jodrell *et al.*, 1991). ZD1694 is principally active through its polyglutamate forms (Jackman *et al.*, 1991) and is currently being clinically evaluated in phase III trials.

In this study, the human lymphoblastoid cell line W1L2 and the human ovarian carcinoma cell line CH1 have been selected for resistance to ZD1694. Using Southern, reverse transcription-polymerase chain reaction and Western techniques we have determined the role of TS expression in determining resistance in these lines. Establishment details and biochemical studies of resistance are described in an accompanying study (Jackman *et al.*, 1995).

### Materials and methods

RPMI-1640 [containing 20 mM 4-(2-hydroxyethyl)-1-piperazine-ethanesulphonic acid buffer and lacking sodium bicarbonate and L-glutamine] was from Flow Laboratories (Irvine, UK). Fetal calf serum was from Imperial Laboratories (Salisbury, UK). Folinic acid (calcium leucovorin 3 mg $ml^{-1}$) was from David Bull laboratories (Warwick, UK). (±)-L-Tetrahydrofolic acid (HCl) (97% pure) was from Fluka (New-Ulm, Germany). [5-$^3$H]dUMP, [$^{32}$P]α-dATP (~30 TBq $mmol^{-1}$) and $^{125}$I-labelled protein A fragments (>1.1 GBq $mg^{-1}$) were supplied by the Radiochemical Centre (Amersham, Buckinghamshire, UK). [6-$^3$H]dUMP was from Moravek Biochemicals (Brea, CA, USA). *Taq* polymerase was purchased from Perkin Elmer Cetus (Norwalk, CT, USA). Moloney murine leukaemia virus (M-MLV) reverse transcriptase and placental ribonuclease inhibitor were from Gibco BRL (Gaithersburg, MD, USA). ZD1694 was synthesised and supplied by ICI Pharmaceuticals PLC (Macclesfield, Cheshire, UK). All other reagents were purchased from Fisons (Loughborough, UK), Sigma (London, UK) or British Drug Houses (BDH) (UK).

Correspondence: J Lunec
*Current address: Massey Cancer Center, Medical College of Virginia, Virginia Commonwealth University, Richmond, Virginia 23298-0037, USA
Received 8 August 1994; revised 21 December 1994; accepted 6 January 1995

*Development of resistant cell lines*

The W1L2:R and CH1:R cell lines were selected by stepwise increases of ZD1694. The selection, culture and pharmacological characterisation of these cell lines are described in an accompanying paper (Jackman et al., submitted for publication). Resistant cells subcultured in ZD1694-containing medium were grown in the absence of ZD1694 for 21 days before all experiments. For the DNA, mRNA and protein determinations, cells were harvested during logarithmic growth and either used immediately or frozen as pellets at −70°C for future use.

*Quantitative Western blotting*

Cell samples were washed twice in phosphate-buffered saline and resuspended at $10^6$ cells per 100 µl of sample buffer (50 mM Tris-Cl pH 6.8, 100 mM dithiothreitol, 1% SDS, 0.2% bromophenol blue and 10% glycerol). Samples were sonicated briefly to shear the DNA, spun for 5 min in a microfuge and the supernatants retained. The crude cell homogenates (10 µl of each) were separated on 12% SDS–polyacrylamide gels and transferred to a nitrocellulose membrane (Burnette, 1981). The membranes were blocked in Tris-buffered saline (TBS) with 0.05% Tween-20 and 5% fat-free milk powder overnight at 4°C. Subsequent hybridisation steps were also carried out in the TBS/Tween/milk powder mixture. The next day the membranes were incubated with a 1:500 dilution of rabbit polyclonal anti-TS antiserum (Freemantle et al., 1991) (kindly provided by Dr Wynne Aherne, Institute of Cancer Research, Sutton, UK). After washing, the filters were incubated in a 1:1000 dilution of $^{125}$I-labelled protein A for 1 h at room temperature. After a final wash to remove excess radiolabel, the membranes were exposed to X-ray film at −70°C with intensifying screens. Bands corresponding to TS were analysed using the PhosphorImaging system to obtain accurate quantification measurements of the relative levels of radioactivity per band.

*cDNA synthesis and quantitative PCR*

The PCR assay is based on the method described by Horikoshi et al. (1992). Total RNA was isolated using the RNAzol method (Cinna/Biotecx Laboratories International, Frienswood, TX, USA; Chomczynski and Sacchi, 1987). cDNA was synthesised from 5 µg of total RNA with 6 µg of random hexamers in a total volume of 100 µl. The reaction mixture consisted of 50 mM Tris–HCl (pH 8.3), 75 mM potassium choloride, 3 mM magnesium chloride, 10 mM dithiothreitol, 1 mM each nucleotide and 600 units of M-MLV reverse transcriptase. The reaction was incubated at 37°C for 1 h.

For the PCR assay the cDNA samples were diluted in sterile water depending on transcript abundance. Three cDNA concentrations for each primer pair were used. For accurate quantification using this method measurements have to be taken in the linear phase of the reaction, where cDNA concentration is directly proportional to signal intensity; using different cDNA concentrations determines whether this region of the reaction curve is covered. PCR amplifications were carried out in a final volume of 25 µl, containing the target cDNA, 12.5 pM of each primer, 2.5 µCi of [α-$^{32}$P]dATP (~30 TBq mM$^{-1}$) and 2 units of *Taq* polymerase in 50 mM Tris–HCl (pH 8.3), 75 mM potassium chloride, 1 mM magnesium chloride and 200 µM each of dCTP, dGTP and dTTP and 100 µM dATP. Each PCR cycle consisted of 1 min of denaturation at 94°C, 1 min of primer annealing at 55°C and 1 min of primer extension at 72°C. A total of 25 cycles were carried out in a Perkin-Elmer/Cetus DNA thermal cycler. Results are expressed as target mRNA levels relative to the internal reference standards: β-actin mRNA and 18S rRNA.

The sequences of the thymidylate synthase (Takeishi et al., 1985) and β-actin (Ng et al., 1985) primers were the same as those used by Horikoshi et al. (1991): TS-5′(5′-AGA TCC-AAC ACA TCC TCC GCT-3′), TS-3′ (5′-CCA GAA CAC ACG TTT GGT TCT CAG-3′), β-actin-5′ (5′-GCG GGA-AAT CGT GCG TGA CAT T-3′) and β-actin-3′ (5′-GAT GGA GTT GAA GGT AGT TTC GTG-3′). The dihydrofolate reductase (DHFR) mRNA (Chen et al., 1984) and 18S rRNA (Torczynski et al., 1985) primers were selected using the primer selection programme designed by Lowe et al. (1990): DHFR-5′ (5′-CCA CAA CCT-CTT CAG TAG AAG-3′), DHFR-3′ (5′-CTT ATT GCC-TTT CTC CTC CTG G-3′), 18S-5′ (5′-GAT GGA GTT GAA GGT AGT TTC GTG-3′) and 18S-3′ (5′-GAA CTA CGA CGG TAT CTG ATC G-3′). All primers were synthesised on an Applied Biosystems 392 DNA/RNA synthesiser.

PCR products were separated on 12% polyacrylamide gels. To 10 µl of each PCR reaction, 3 µl of 5 × sample loading buffer was added (10% Ficoll, 0.05% bromophenol blue, 0.25% orange G and 0.5% SDS in water). The acrylamide gels were dried down and radioactive PCR product bands were located by autoradiography for excision and subsequent quantification by liquid scintillation counting. Background control bands from each lane were also excised, and these counts were subtracted from the PCR product band counts. Transcript abundance was calculated by relating target mRNA levels, TS and DHFR, to the internal reference standard levels, β-actin mRNA and 18S RNA.

*DNA analysis*

DNA extractions were carried out either using the Applied Biosystems 340A nucleic acid extractor (Applied Biosystems, CA, USA) or manually. The initial stages for each method are identical. Cell membranes were disrupted by gently mixing the cells with nuclear isolation buffer [NIB: 0.25% of Nonidet P40 (non-ionic detergent), 100 mM sodium chloride, 10 mM Tris–HCl, 1 mM EDTA]. Approximately 2 ml of NIB was used per $10^8$ cells. The nuclei were then spun down for 5 min at 2000 *g* (4°C) and the supernatant removed. The nuclear pellets were resuspended in the same volume of NIB as before plus a further volume of 2 × lysis buffer (Applied Biosystems product: urea, sodium chloride, *n*-lauroyl sarcosine and 1,2-cyclohexaminediamine tetra-acetic acid in Tris–HCl, pH 7.9) and proteinase K added to a final concentration of 600 µg ml$^{-1}$. This mixture was incubated at 60°C for 2 h or until the majority of the protein was digested. For the automated process the proteinase K digest is loaded onto the machine which carries out automated phenol–chloroform extractions and isopropanol precipitations. The final DNA precipitate is collected onto a filter from which it can be redissolved in the required solution. For the manual DNA procedure organic extractions were carried out essentially as described by Sambrook et al. (1989).

For Southern transfer, DNA samples were digested with the restriction endonuclease *Eco*RI, and the fragments separated by electrophoresis on 0.8% agarose gels before transfer to and immobilisation on nylon membranes. Probe hybridisation was carried out under standard conditions at 65°C with a final wash stringency of 2 × standard saline citrate (1 × = 0.15 M sodium chloride, 0.015 M sodium citrate, pH 7.0) and 0.2% sodium dodecyl sulphate (SDS). The probe used for TS DNA and mRNA analysis was a 0.7 kb gel-purified fragment of mouse TS cDNA cleaved from the pMTS-3 plasmid with *Hin*dIII and *Pst*I (Geyer and Johnson, 1984) which was $^{32}$P labelled by random primer extension (Feinberg and Vogelstein, 1983). Equal loading of DNA was determined by ethidium bromide staining of the gels before transfer. A non-amplified cross-reacting sequence was evident in all the samples probed with the TS cDNA probe, and this was also used to check for equal DNA loading. This sequence has been previously reported (Berger et al., 1985; Clark et al., 1987) with the suggestion that this sequence is a TS pseudogene which exists at an alternative chromosomal location (Clark et al., 1987). To estimate the degree of TS gene amplification, dilutions of the resistant cell line DNA and RNA samples were made to obtain a signal of equal intensity to that produced by undiluted parental DNA. The membranes were exposed to X-ray film at −70°C with intensifying screens and for quantification of signals the filters were analysed using the PhosphorImager system (Molecular Dynamics, Sunnyvale, CA, USA).

### Results

For detailed cell characterisation see the accompanying paper by Jackman *et al.* in this issue. Briefly, the W1L2:R cell line was >20 000-fold resistant and the CH1:R cell line was approximately 14-fold resistant to ZD1694. There was an increase in TS activity in both cell lines (W1L2:R, 514-fold; and CH1:R, 4.2-fold). There were no significant differences in DHFR activity.

### *Protein analysis*

The Western blots in Figure 1 demonstrate the different levels of TS protein in the resistant *vs* parental cell lines. Using the PhosphorImager the relative amount of $^{125}$I-labelled protein A was quantified in the bands corresponding to the molecular weight of the human TS monomer (36 kDa). The results from this analysis and from two repeat experiments show that the total TS protein level in CH1:R cells was approximately 2.5-fold higher than that of the parental cell line. TS protein determinations using the W1L2 and W1L2:R cell lines (Figure 1b) indicated that the level of TS monomer in the resistant cells was elevated by approximately 1000-, 600- and 500-fold from three separate experiments.

### *DNA analysis*

Figure 2 shows the Southern analysis for TS with DNA from the W1L2 and W1L2:R cell lines. The PhosphorImager was used to quantify the relative amounts of radioactivity in each band and the difference in TS gene copy number between the cell lines was calculated. The increase in TS gene copy number in the W1L2:R compared with the W1L2 cell line, calculated from this blot, was 114-fold with a 95% confidence interval of 66- to 162-fold ($P = 0.009$). In two repeats of this analysis (not shown) the increase in TS gene copy number in the W1L2:R cell line appeared to be approximately 60- to 100-fold.

Figure 3 shows the Southern analysis for TS gene copy levels for the CH1 and CH1:R cell line. The 4.2-fold elevation in TS catalytic activity in the CH1:R cell line indicated that if gene amplification were the cause of this overexpression the increase in gene copy number would be small. Using the PhosphorImager the fold increase in TS gene copy number in the CH1:R compared with the CH1 cell line was 2.6-fold with a 95% confidence interval of 1.8- to 3.3-fold ($P =$ 0.0031 with five degrees of freedom). Two repeat experiments of this Southern analysis also indicated an approximately 2-fold increase in TS gene copy number in the CH1:R compared with the CH1 cell line.

### *RNA analysis*

Table I shows the results from PCR-based transcription assays using W1L2, W1L2:R, CH1, and CH1:R cell line cDNAs. The TS mRNA levels are clearly elevated in the W1L2:R line compared with the parental W1L2 cell line, the average difference between the two varying from 82-fold relative to β-actin mRNA to 128-fold relative to 18S RNA. The difference between these two values is explained when the β-actin mRNA relative to 18S rRNA expression ratios for the two cell lines are examined: there is a significant increase in this ratio in the resistant cell line compared with the parental line (1.4- to 1.5-fold). This may indicate an increase in the total mRNA transcription level which is either essential for, or a consequence of, the drug resistance phenotype.

The reported increase in the TS catalytic activity of the CH1:R cell line was 4.2-fold and the observed increase in TS gene copy number from the previous section was approximately 2-fold. A 1.9-fold increase in TS mRNA relative to β-actin mRNA was seen in the CH1:R line compared with the CH1 line, which reached significance in a paired two-tailed *t*-test ($P = 0.0378$). A similar increase was seen when TS mRNA levels were expressed relative to DHFR mRNA and 18S rRNA levels, but this did not achieve significance. There was no significant difference in the β-actin/18S expression ratios between the CH1 and CH1:R cell lines.

Northern analysis using total RNA from these cell lines (not shown) suggested that there was a small increase (approximately 2-fold) in TS mRNA levels in the resistant cell line. The sum of the mean values from the three TS expression ratios (relative to DHFR, β-actin and 18S) indicates that the increase in TS mRNA level, although small (approximately 2-fold), does achieve statistical significance ($P = 0.0179$).

**Figure 1** Western analysis of TS in (**a**) CH1 and CH1:R and (**b**) W1L2 and W1L2:R total cellular protein. The gels were blotted and the membranes probed with the TS polyclonal antiserum. TS monomer molecular weight is ~36 kDa. (**a**) Lane 1 contains 5 ng of human recombinant TS, lanes 2–4 contain CH1 protein and lanes 5–9 contain CH1:R protein. (**b**) Lane 1 contains 5 ng of human recombinant TS, lanes 2–4 contain W1L2 protein and lanes 5–8 contain W1L2:R protein.

**Figure 2** Southern analysis of the TS gene from W1L2 and W1L2:R DNA (*Eco*RI digest). Lanes 1 and 2 contain 10 and 5 μg of W1L2 DNA, respectively, and lanes 3–8 contain 5, 2.5, 1.25, 0.63, 0.32 and 0.15 μg of W1L2:R DNA respectively.

**Figure 3** Southern analysis of the TS gene from CH1 and CH1:R DNA (*Eco*RI digest). Lanes 1 and 2 contain 20 and 10 μg of CH1 DNA, respectively, and lanes 3–6 contain 20, 10, 5 and 2.5 μg of CH1:R DNA respectively.

**Table I** Results from quantitative PCR-based transcription assays comparing TS, DHFR, β-actin mRNA and 18S rRNA expression ratios between the parental W1L2 and CH1 cell lines and the ZD1694-resistant variants

| *Expression ratio* | *TS/β-action (× 10⁻³)* | *TS/DHFR* | *TS/18S (× 10⁻⁶)* | *DHFR/β-actin* | *DHFR/18S (× 10⁻³)* | *β-actin/18S (× 10⁻³)* |
|---|---|---|---|---|---|---|
| W1L2 | | | | | | |
| *n* | 5 | 2 | 5 | 2 | 2 | 5 |
| Mean | 28 | 0.47 | 31 | 0.055 | 0.080 | 1.23 |
| s.d. | 11 | 0.23 | 6.3 | 0.023 | 0.033 | 0.35 |
| W1L2:R | | | | | | |
| Mean | 2300 | 48 | 3900 | 0.046 | 0.093 | 1.73 |
| s.d. | 860 | 31 | 1850 | 0.017 | 0.022 | 0.48 |
| Mean (fold-change) ± s.e. | 89 ± 38 | 91 ± 20 | 122 ± 39 | 0.87 ± 0.07 | 1.3 ± 0.25 | 1.5 ± 0.40 |
| *P*-value paired two-tailed *t* test | 0.0045 | | 0.0134 | | | 0.048 |
| CH1 | | | | | | |
| *n* | 6 | 3 | 6 | 3 | 3 | 6 |
| Mean | 33 | 0.33 | 64 | 0.13 | 0.27 | 2.1 |
| s.d. | 17.5 | 0.067 | 29 | 0.031 | 0.020 | 0.78 |
| CH1:R | | | | | | |
| Mean | 53 | 0.53 | 132 | 0.131 | 0.20 | 2.9 |
| s.d. | 20 | 0.26 | 86 | 0.098 | 0.12 | 2.7 |
| Mean (fold-change) ± s.d. | 1.85 ± 0.79 | 1.7 ± 0.74 | 2.7 ± 2.4 | 0.88 ± 0.47 | 0.76 ± 0.49 | 1.2 ± 0.64 |
| *P*-value, paired two-tailed *t* test | 0.038 | 0.22 | 0.178 | 0.96 | 0.54 | 0.46 |

Fold-change is defined as ($value_{resistant}/value_{parental}$) for each individual determination. *n* refers to the number of determinations for each resistant and parental cell line pair.

**Table II** Results summary of the increases in TS gene copy number, mRNA and protein levels, and fold resistance to the selective agent in the W1L2:R and CH1:R cell lines. Also shown for comparison are previously reported data for the W1L2:C1 cell line (O'Connor *et al.*, 1992)

| *Approximate fold increase from parental cell line in* | *W1L2:R* | *CH1:R* | *W1L2:C1* |
|---|---|---|---|
| TS gene copy number | ~100 | 2-2.5 | 64-96 |
| TS mRNA levels | 82-128 | 2 | 86-135 |
| TS protein levels | 500-1000 | 2.5 | 125-150 |
| Resistance to selecting agent | >20 000 | 14 | 27 000 |

For both cell lines there was no significant difference in the DHFR/β-actin or DHFR/18S expression ratios between the parental cell line and the corresponding resistant variant.

The results from the W1L2:R and CH1:R cell lines are summarised in Table II. In both of the cell lines studied, the increase in the level of resistance obtained to the selective agent exceeds the increases in the TS gene copy number, mRNA, protein or catalytic activity levels determined. In the CH1:R cell line the increase in TS gene copy number was associated with a similar increase in TS mRNA and protein; in the W1L2:R cell line the increase in TS protein levels and activity exceeds that of the increase in gene copy and mRNA levels by at least 4-fold.

### Discussion

Two cell lines selected for the ability to grow in high levels of the folate-based TS inhibitor, ZD1694, were studied to determine their mechanisms of resistance. Initial studies revealed increases in TS catalytic activity in both cell lines, and we found that this was associated with TS gene amplification and overexpression and not with an alteration in the enzyme itself. Neither of the resistant cell lines showed evidence of gene rearrangement involving the *Eco*RI fragment detected by Southern blot analysis. This, together with the proportionate increase in TS mRNA levels and maintenance of the normal transcript sizes in the cell lines, strongly suggested that the amplified TS genes were intact and transcriptionally active. The regions of DNA which contain amplified genes (amplicons) for other genetic markers are reported to range in size from approximately 50 kb to >10 Mb, which could comfortably contain the full 16 kb of the biologically active TS sequence (Kaneda *et al.*, 1990).

The most common biochemical alterations associated with acquired resistance to the antifolate methotrexate (MTX) are overexpression of the target enzyme DHFR (Biedler and Spengler, 1975, 1976; Kaufman, 1979; Flintoff *et al.*, 1982), altered drug transport (Schuetz *et al.*, 1988; Norris *et al.*, 1991; Trippett *et al.*, 1992) and altered drug polyglutamation (McCloskey *et al.*, 1991; Van der Laan *et al.*, 1991). This pattern would appear to apply equally to the folate-based TS inhibitor, ZD1694; cell lines selected for resistance to ZD1694 have also demonstrated these three manifestations of resistance (accompanying study; Jackman *et al.*, 1995).

There were no significant differences in DHFR enzyme activity or transcript levels between the parental cells and the ZD1694-resistant variants. This contrasts with the murine L1210:C15 cell line, selected for a high level of resistance to the TS inhibitor CB3717, which has been reported to have approximately 45-fold increased TS gene copy number and mRNA levels plus a 30- to 40-fold increase in DHFR gene copy number with a 7-fold elevation of DHFR mRNA levels (Imam *et al.*, 1987). As DHFR was not overexpressed in either cell line selected for resistance to ZD1694, it does not seem to be a prerequisite for cell lines resistant to high levels of folate-based TS inhibitors to overexpress the DHFR gene with the TS gene.

In both of the cell lines described in this study the fold resistance exceeds the fold increase in TS gene copy number, mRNA and protein levels. Possible explanations relating to altered transport or polyglutamination are addressed in an accompanying study (Jackman *et al.*, 1995). The CH1:R cell line showed similar elevations of TS gene copy number, mRNA levels, catalytic activity and protein, whereas in the W1L2:R cell line the fold increase in enzyme activity and TS monomer was greater than that of TS gene copy number or mRNA. This suggests an alteration in the level of translation of TS specific mRNA in W1L2:R cells which could be due to changes in the transcribed non-coding regions of the amplified gene, in a translational regulatory protein or even associated with the probable extrachromosomal location of the amplified TS genes in this cell line. It has been noted in three recent reports that translational mechanisms are involved in the regulation of TS expression (Kaneda et al., 1987; Chu et al., 1991; Keyomarsi et al., 1993).

Differences in the naturally occurring levels of TS and any changes in these levels subsequent to drug administration may be critical determinants of clinical response to these agents. Keyomarsi et al. (1993) have reported that, following ZD1694 treatment, TS activity, but not mRNA levels, increases by up to 40-fold in normal and 10-fold in tumour-derived cell lines, suggesting a loss of TS translational regulation in the presence of ZD1694. The importance of TS levels to the cytotoxicity of these compounds *in vitro* is demonstrated by the cell lines described in this report. For future consideration it will be of interest to relate innate TS levels to the activity of these compounds *in vitro*, *in vivo* and in the clinic.

**Abbreviations:** 5,10-$CH_2$-$FH_4$, 5,10-methylene tetrahydrofolic acid; 5-FU, 5-fluorouracil; CB3717, $N^{10}$-propargyl-5,8-dideazafolic acid; cDNA, complementary DNA; D1694, N-(5-[*N*-(3,4-dihydro-2-methyl-4-oxoquinazolin-6-ylmethyl)-*N*-methyl- amino]-2-thenoyl)-L-glutamic acid; dATP, deoxyadenosine triphosphate; DHFR, dihydrofolate reductase; dUMP, deoxyuridine monophosphate; dTMP, thymidine monophosphate; EDTA, ethylenediamine-tetraacetic acid; FdUrd, 5-fluorodeoxyuridine; FdUMP, 5-fluorodeoxyuridine monophosphate; FPGS, folylpolyglutamate synthetase; ICI198583, $C^2$-desamino-$C^2$-methyl-$N^{10}$-propargyl-5,8-dideazafolic acid; PCR, polymerase chain reaction; TS, thymidylate synthase.

**Acknowledgements**

This work was supported by a grant from the North of England Cancer Research Campaign.

### References

BASSENDINE MF, CURTIN NJ, LOOSE H, HARRIS AL AND JAMES OFW. (1987). Indication of remission in hepatocellular carcinoma with a new thymidylate synthase inhibitor CB3717: a phase II study. *J. Hepatol.*, **4**, 349–356.

BERGER SH, JENH C-H, JOHNSON LF AND BERGER FG. (1985). Thymidylate synthase overproduction and gene amplification in fluorodeoxyuridine-resistant human cells. *Mol. Pharmacol.*, **28**, 461–467.

BIEDLER JL AND SPENGLER BA. (1975). A novel chromosome abnormality in human neuroblastoma and antifolate-resistant Chinese hamster cells in culture. *J. Natl Cancer Inst.*, **57**, 683–695.

BIEDLER JL AND SPENGLER BA. (1976). Metaphase chromosome anomaly: association with drug resistance and cell specific products. *Science*, **191**, 185–187.

BURNETTE WN (1981). 'Western blotting': electrophoretic transfer of proteins from sodium dodecyl sulphate–polyacrylamide gels to unmodified nitrocellulose and radiographic detection with antibody and radioiodinated protein A. *Anal. Biochem.*, **112**, 195–203.

CALVERT AH, ALISON DL, HARLAND SJ, ROBINSON BA, JACKMAN AL, JONES TR, NEWELL DR, SIDDIK ZH, WILTSHAW E, McELWAIN TJ, SMITH IE AND HARRAP KR. (1986). A phase I evaluation of the quinazoline antifolate thymidylate synthase inhibitor, $N^{10}$-propargyl-5,8-dideazafolic acid, CB3717. *J. Clin. Oncol.*, **4**, 1245–1252.

CHEN MJ, SHIMADA T, MOULTON AD, CLINE A, HUMPHRIES RK, MAIZEL J AND NIENHUIS AW. (1984). The functional human dihydrofolate reductase gene. *J. Biol. Chem.*, **259**, 3933–3943.

CHOMCZYNSKI P AND SACCHI N. (1987). Single-step method of RNA isolation by acid guanidinium thiocyanate–phenol–chloroform extract. *Anal. Biochem.*, **162**, 156–159.

CHU E, KOELLER DM, CASEY JL, DRAKE JC, CHABNER BA, ELWOOD PC, ZINN S AND ALLEGRA CJ. (1991). Autoregulation of human thymidylate synthase messenger RNA translation by thymidylate synthase. *Proc. Natl Acad. Sci. USA*, **88**, 8977–8981.

CLARK JL, BERGER SH, MITTELMAN A AND BERGER FG. (1987). Thymidylate synthase gene amplification in a colon tumour resistant to fluoropyrimidine chemotherapy. *Cancer Treat. Rep.*, **71**, 261–265.

FEINBERG AP AND VOGELSTEIN B. (1983). A technique for radiolabelling DNA restriction endonuclease fragments to high specific activity. *Anal. Biochem.*, **132**, 6–13.

FLINTOFF WF, WEBER MK, NAGAINIS CR, ESSANI AK, ROBERTSON D AND SALSER W. (1982). Overproduction of dihydrofolate reductase and gene amplification in methotrexate-resistant Chinese hamster ovary cells. *Mol. Cell. Biol.*, **2**, 275–285.

FREEMANTLE SJ, AHERNE GW, HARDCASTLE A, LUNEC J AND CALVERT AH. (1991). Increases in thymidylate synthase protein levels measured using newly developed antibodies. *Proc. Am. Assoc. Cancer Res.*, **32**, 360.

GEYER PK AND JOHNSON LF. (1984). Molecular cloning of DNA sequences complementary to mouse thymidylate synthase messenger RNA. *J. Biol. Chem.*, **259**, 7206–7211.

GLAZER RI AND LLOYD LS. (1982). Association of cell lethality with incorporation of 5-fluorouracil and 5-fluorouridine into nuclear RNA in human carcinoma cells in culture. *Mol. Pharmacol.*, **21**, 468–473.

HERRICK D AND KUFE DW. (1984). Lethality associated with incorporation of 5-fluorouracil into preribosomal RNA. *Mol. Pharmacol.*, **26**, 135–140.

HORIKOSHI T, DANENBERG KD, STADLBAUER THW, VOLKENANDT M, SHEA LC, AIGNER K, GUSTAVSSON B, LEICHMAN L, FROSING R, RAY M, GIBSON NW, SPEARS CP AND DANENBERG PV. (1992). Quantitation of thymidylate synthase, dihydrofolate reductase, and DT-diaphorase gene expression in human tumours using the polymerase chain reaction. *Cancer Res.*, **52**, 108–116.

IMAM AM, CROSSLEY PH, JACKMAN AL AND LITTLE PFR. (1987). Analysis of thymidylate synthase gene amplification and of mRNA levels in the cell cycle. *J. Biol. Chem.*, **262**, 7368–7373.

JACKMAN AL, TAYLOR GA, GIBSON W, KIMBELL R, BROWN M, CALVERT AH, JUDSON IR AND HUGHES LR. (1991). ICI D1694, a quinazoline antifolate thymidylate synthase inhibitor that is a potent inhibitor of L1210 tumor cell growth *in vitro* and *in vivo*: a new agent for clinical study. *Cancer Res.*, **51**, 5579–5586.

JACKMAN AL, KELLAND LR, BROWN M, GIBSON W, KIMBELL R, AHERNE W AND JUDSON IR. (1992). ICI D1694 resistant cell lines. *Proc. Am. Assoc. Cancer Res.*, **33**, 406.

JACKMAN AL, KELLAND LR, KIMBELL R, BROWN M, GIBSON W, AHERNE GW, HARDCASTLE A AND BOYLE FT. (1995). Mechanisms of acquired resistance to the quinazoline thymidylate synthase inhibitor, ZD1694 (Tomudex) in one mouse and three human cell lines. *Br. J. Cancer*, **71**, 914–924.

JENH C-H, GEYER PK, BASKIN F AND JOHNSON LF. (1985). Thymidylate synthase gene amplification in fluorodeoxyuridine-resistant mouse cell lines. *Mol. Pharmacol.*, **28**, 80–85.

JODRELL DI, NEWELL DR, MORGAN SE, CLINTON S, BENSTED JP, HUGHES LR AND CALVERT AH. (1991). The renal effects of N10-propargyl-5,8-dideazafolic acid (CB3717) and a non-nephrotoxic analogue ICI D1694, in mice. *Br. J. Cancer*, **64**, 833–838.

JONES TR, CALVERT AH, JACKMAN AL, BROWN SJ, JONES M AND HARRAP KR. (1981). A potent antitumour quinazoline inhibitor of thymidylate synthase: synthesis, biological properties and therapeutic results in mice. *Eur. J. Cancer*, **17**, 11–19.

KANEDA S, TAKEISHI K, AYUSAWA D, SHIMUZU K, SENO T, AND ALTMAN S. (1987). Role in translation of a triple tandemly repeated sequence in the 5′-untranslated region of the human thymidylate synthase mRNA. *Nucleic Acids Res.*, **15**, 1259–1270.

KANEDA S, NALBANTOGLU J, TAKEISHI K, SHIMUZU K, GOTOH O, SENO T AND AYUSAWA D. (1990). Structural and functional analysis of the human thymidylate synthase gene. *J. Biol. Chem.*, **265**, 20277–20284.

KAUFMAN RJ, BROWN PC AND SCHIMKE RT. (1979). Amplified dihydrofolate reductase genes in unstably methotrexate-resistant cells are associated with double minute chromosomes. *Proc. Natl Acad. Sci. USA*, **76**, 5669–5673.

KEYOMARSI K, SAMET J, MOLNAR G AND PARDEE AB. (1993). The thymidylate synthase inhibitor, ICI-D1694, overcomes translational detainment of the enzyme. *J. Biol. Chem.*, **268**, 15142–15149.

KUFE DW AND MAJOR PP. (1981). 5-Fluorouracil incorporation into human breast carcinoma RNA correlates with cytotoxicity. *J. Biol. Chem.*, **256**, 9802–9805.

KUFE DW, MAJOR PP, EGAN EM AND LOH E. (1981). 5-Fluoro-2′-deoxyuridine incorporation in L1210 DNA. *J. Biol. Chem.*, **256**, 8885–8888.

LOWE T, SHAREFKIN J, YANG SQ AND DIEFFENBACH CW. (1990). A computer program for selection of oligonucleotide primers for polymerase chain reactions. *Nucleic Acids Res.*, **18**, 1757–1761.

McCLOSKEY DE, McGUIRE JJ, RUSSELL CA, ROWAN BG, BERTINO JR, PIZZORNO G AND MINI E. (1991). Decreased folylpolyglutamate synthetase activity as a mechanism of methotrexate resistance in CCRF-CEM human leukemia sublines. *J. Biol. Chem.*, **266**, 6181-6187.

NG S-Y, GUNNING P, EDDY R, PONTE P, LEAVITT J, SHOWS T AND KEDES L. (1985). Evolution of the functional human β-actin gene and its multi-pseudogene family: conservation of noncoding regions and chromosomal dispersion of pseudogenes. *Mol. Cell Biol.*, **5**, 2720-2732.

NORRIS MD, HABER M, KAVALLARIS M, BRIAN MJ, LUTZE LH, WHITE L AND STEWART BW. (1991). Reduced drug accumulation as the mechanism of extreme clinical resistance to methotrexate in the human T-cell leukemia xenograft, LALW-2. *Cancer*, **68**, 981-987.

O'CONNOR BM, JACKMAN AL, CROSSLEY PH, FREEMANTLE SJ, LUNEC J AND CALVERT AH. (1992). Human lymphoblastoid cells with acquired resistance to $C^2$-desamino-$C^2$-methyl-$N^{10}$-propargyl-5,8-dideazafolic acid: a novel folate-based thymidylate synthase inhibitor. *Cancer Res.*, **52**, 1137-1143.

PRIEST DG AND LEDFORD BE. (1980). Increased thymidylate synthetase in 5-fluorodeoxyuridine resistant cultured hepatoma cells. *Biochem. Pharmacol.*, **29**, 1549-1553.

SAMBROOK J, FRITSCH EF AND MANIATIS T. (1989). *Molecular Cloning: A Laboratory Manual*, 2nd edn. Cold Spring Harbor Press: Cold Spring Harbor, NY.

SAWYER RC, STOLFI RL, MARTIN DS AND SPIEGELMAN S. (1984). Incorporation of 5-fluorouracil into murine bone marrow DNA *in vivo*. *Cancer Res.*, **44**, 1847-1851.

SCHUETZ JD, MATHERLY LH, WESTIN EH AND GOLDMAN ID. (1988). Evidence for a functional defect in the translocation of the methotrexate transport carrier in a methotrexate-resistant murine L1210 leukemia cell line. *J. Biol. Chem.*, **263**, 9840-9847.

SESSA C, ZUCCHETTI GINIER M, WILLEMS Y, D'INCALCI M AND CAVALLI F. (1988). Phase I studies of the antifolate $N^{10}$-propargyl-5,8-dideazafolic acid, CB3717. *Eur. J. Cancer Clin. Oncol.*, **24**, 769-775.

TAKEISHI K, KANEDA S, AYUSAWA D, SHIMIZU K, GOTOH O AND SENO T. (1985). Nucleotide sequence of a functional cDNA for human thymidylate synthase. *Nucleic Acids Res.*, **13**, 2035-2043.

TORCZYNSKI RM, FUKE M AND BOLLON AP. (1985). Cloning and sequencing of a human 18S ribosomal RNA gene. *DNA*, **4**, 283-291.

TRIPPETT T, SCHLEMMER S, ELISSEYEFF Y, GOKER E, WACHTER M, STEINHERZ P, TAN C, BERMAN E, WRIGHT JE, ROSOWSKY A AND OTHERS. (1992). Defective transport as a mechanism of acquired resistance to methotrexate in patients with acute lymphocytic leukemia. *Blood*, **80**, 1158-1162.

VAN DER LAAN BFAM, JANSEN G, KATHMANN I, SCHORNAGEL JH AND HORDIJK GJ. (1991). Mechanisms of acquired resistance to methotrexate in a human squamous carcinoma cell line of the head and neck, exposed to different drug schedules. *Eur. J. Cancer*, **27**, 1274-1278.

WILKINSON DS, TLSTY TD AND HANAS RJ. (1975). The inhibition of ribosomal RNA synthesis and maturation in Novikoff hepatoma cells by 5-fluorouridine. *Cancer Res.*, **35**, 3014-3020.